# A systematic review of unmet needs of older adults in home settings and their implications for novel technological solutions

**DOI:** 10.1093/geroni/igaf106

**Published:** 2025-10-10

**Authors:** Andrew Dolman, Sidharth Kaliappan, Yanling Zhou, Divija Palleti, Jenna Marquard, Sunghoon Ivan Lee, Ravi Karkar, Holly Brugge Jimison

**Affiliations:** Bouvé College of Health Sciences, Northeastern University, Boston, Massachusetts, United States; Manning College of Information and Computer Sciences, University of Massachusetts Amherst, Amherst, Massachusetts, United States; Bouvé College of Health Sciences, Northeastern University, Boston, Massachusetts, United States; Khoury College of Computer Science, Northeastern University, Boston, Massachusetts, United States; Manning College of Information and Computer Sciences, University of Massachusetts Amherst, Amherst, Massachusetts, United States; School of Nursing, University of Minnesota, Minneapolis, Minnesota, United States; Manning College of Information and Computer Sciences, University of Massachusetts Amherst, Amherst, Massachusetts, United States; Manning College of Information and Computer Sciences, University of Massachusetts Amherst, Amherst, Massachusetts, United States; Khoury College of Computer Science, Northeastern University, Boston, Massachusetts, United States

**Keywords:** Aging in place, Needs assessment, Thematic analysis, Artificial intelligence

## Abstract

**Background and Objectives:**

To better support aging in place, we first must understand the needs of the older adult population. We conducted a systematic review to understand the needs of older adults in the home.

**Research Design and Methods:**

We queried the PubMed, CINAHL, and ProQuest databases to identify literature related to needs assessments of older adults in the home. Records were included if: (1) the population focused on older adults (aged 65 years and older); (2) a needs assessment was conducted; (3) the older adult population was aging in place and not in a long-term care facility; (4) English language publication; (5) published since 2013; and (6) pertaining solely to older adult caregivers’ needs. The needs identified in each article were extracted and categorized based on emergent themes.

**Results:**

A total of 1,963 records were identified. After removing duplicate records and those not meeting the inclusion criteria, 65 articles were included in the final analysis. Six need-related theme domains were identified: health management needs; social needs; homecare and practical needs; information needs; technology needs; and healthcare system needs.

**Discussion and Implications:**

Through the systematic review, we identified a wide range of unmet needs for older adults aging in the home. The unmet needs of older adults are multifaceted and provide ideal targets for the development of novel technological solutions. In particular, recent advances in artificial intelligence (AI), especially generative AI such as large language models (LLMs), surface the potential for technology to address unmet needs across multiple domains. We discuss the potential for AI to lower barriers to technology uptake for older adults and create novel solutions to each of the need domains identified. Ultimately, AI-enabled solutions may increase independence for older adults and potentially increase the ability to age in place.

Translational Significance: In this systematic review, we identified the unmet needs of older adults who are aging in place. Our findings span across multiple needs domains–health management, socialization, homecare, information access, technology use, and interacting with the healthcare system. The unmet needs we identified provide opportunities for meaningful technological solutions to support older adults. Our review surfaced articles that suggested older adults are open to using technology but face barriers to use (eg, limited skills, high costs, unsuitable devices). We argue that solutions using artificial intelligence, including large language models, may reduce barriers and support targeting unmet needs in novel ways.

## Introduction

The global population is aging, with the number of adults aged 60 years and above expected to reach 2.1 billion by 2050.^1^ To support healthy aging for individuals in this growing demographic, it is critical to understand their unmet needs. One key component of healthy aging is the ability for older adults to age in place within their communities and homes, rather than in long-term care facilities. Older adults prefer living independently within their homes, feel a greater sense of autonomy, and this model is far less expensive than living in long-term care facilities.^2^^,^^3^ Unmet needs that hinder older adults’ ability to age in their homes need to be identified and addressed.

Recent systematic reviews of the literature have provided partial insights into the unmet needs of older adults.^4^^,^^5^ The reviews typically focused on either (1) confirming or expanding on a pre-defined need, (2) identifying causes or effects of a pre-defined need, or (3) needs of a specific sub-population of older adults. For example, one recent review focused on unmet nursing care-specific needs of older adults receiving nursing care in the home, hospital, and long-term care facilities.^4^ Another systematic review focused on the link between unmet needs specific to activities of daily living (ADLs), healthcare utilization, as well as depression and anxiety levels.^5^ This systematic review adds to the literature by identifying older adults’ unmet needs specific to aging in the home through a broader lens without a focus on specific sub-populations. The review focuses on the general older adult population and offers a more inclusive understanding of the diverse needs of older adults.

### Research question

This systematic review aimed to identify literature to answer the research question: “*What are the unmet needs of older adults who are aging in place?*”

## Methods

### Database queries

Three databases, PubMed, CINAHL, and ProQuest, were queried in October 2023. Databases were queried to identify literature (peer-reviewed journals, conference proceedings, news articles, books) pertaining to older adults not living in long-term care facilities such as nursing homes. The search strategy combined booleans, truncation, MeSH terms, CINAHL headings, and the ProQuest Thesaurus to create a broad literature search. Details of the search terms for each database are provided in **Supplementary Materials**.

### Screening records

Titles and abstracts were extracted from the search results and loaded into EndNote for deduplication. The research team screened the articles based on the following inclusion criteria: (1) the population focused on older adults (aged 65 years and older); (2) a needs assessment was conducted–defined as identifying or evaluating gaps in required support, services, or resources based on self-report or secondary analyses; (3) the older adult population was aging in place and not in a long-term care facility such as a nursing home; (4) English language publication; (5) published since 2013; and (6) pertaining solely to older adult caregivers’ needs. Full-text articles were obtained for all records meeting the inclusion criteria. After review of the full text articles, records that did not meet all inclusion criteria were removed. Decisions to include or exclude records were reviewed during weekly research team meetings. Differences in opinion on inclusion/exclusion were decided through majority consensus of senior research team members (ie, agreement between at least three of four members).

### Data extraction

Details from the full text articles were extracted by the research team into an Airtable database—a cloud-based relational database conducive to multisite collaboration that enabled structured data entry, customizable fields, efficient organization of themes, and version control with real-time updates. Article details included author, publication year, population, average age, gender distribution, sample size, country, research methodology, and needs findings. Additionally, articles were categorized as original needs assessments if the needs’ findings were obtained from primary self-report, and articles were categorized as prior research if the findings were obtained through secondary analysis or systematic review that summarized unmet needs in previously published research. Thematic analysis was used to categorize older adult needs into distinct theme groups that emerged as findings were iteratively reviewed throughout the coding process.

## Results

**Figure 1.**
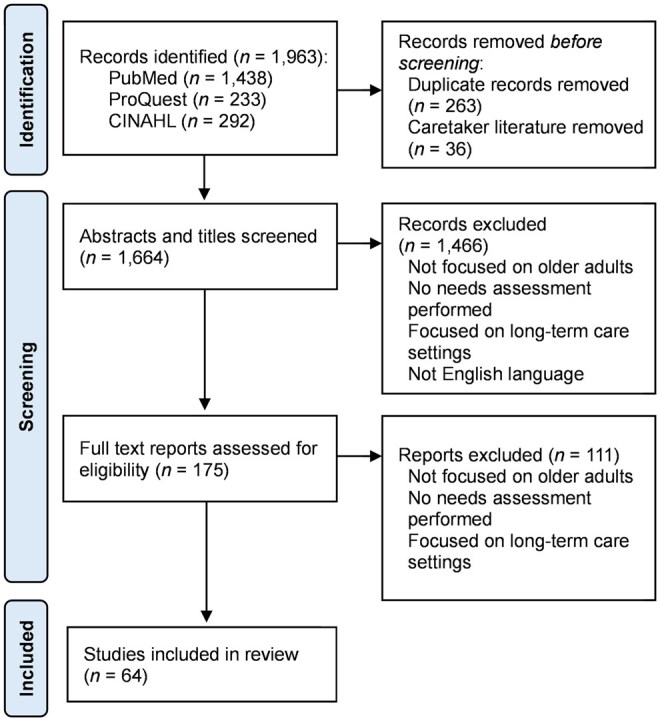
PRISMA 2020 flow diagram. Note. Articles were obtained across three databases and screened for eligibility. The systematic review included 64 articles in the final analysis.

A total of 1,963 records were identified after querying the three databases (PubMed *n *= 1,438; ProQuest *n *= 233; CINAHL *n *= 292). There were 263 duplicate records removed from the search results, and 36 records removed because they focused solely on older adult caregiver needs. Abstracts and titles were screened for 1,664 records, and 1,466 records were excluded. Full-text articles were obtained for 175 records. After reviewing full-text articles, 111 articles were removed, and 64 articles were included in this systematic review: 40 articles conducted primary original needs assessments, and 24 articles included prior research based on secondary analyses or systematic reviews. The PRISMA 2020 flow diagram of this systematic review is shown in Figure 1.

**Figure 2.**
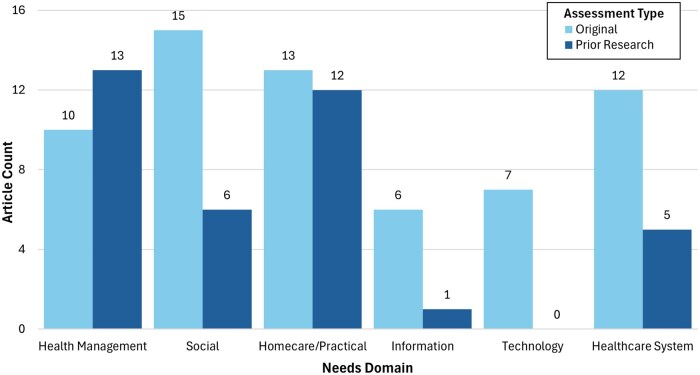
Article counts by needs domain and assessment type. Note. Article counts are presented by the unmet needs thematic domain identified during the systematic review. The counts are stratified by the assessment type: the number of original needs assessment articles and the number of presumed needs articles.

The research team reviewed rounds of grouped need themes to ultimately form six domains: health management needs; social needs; homecare and practical needs; information needs; technology needs; and healthcare system needs (see Figure 2 for a count of research articles per domain). Each domain contained subcategories with more specific needs related to that domain (see Table 1 for a summary of unmet needs and examples of unmet needs by subtheme). The identified domains are not mutually exclusive, and needs may span multiple domains due to the multidimensional nature of unmet needs.

**Table 1.** Summary of identified unmet needs.

Theme	Subtheme	Example of need
**Health management needs**	Physical health needs	Increasing physical activity Improving frailty Weight loss and weight management Oral health and tooth loss prevention Eye health and vision impairments Improving mobility and preventing falls
	Cognitive and mental health needs	Managing memory problems and cognitive health Psychological consulting and rest
	Chronic condition management needs	Managing symptoms of chronic illnesses Managing pain
**Social needs**	Social connections	Feelings of isolation and loneliness Providing feeling of belonging Improving social cohesion Satisfaction with relationships and social life Opportunities for socialization
	Emotional support needs	Support for emotional needs Availability to talk to someone about feelings Coping with feelings of increased dependency Improving self-esteem Reducing hopelessness
**Homecare/ ** **practical needs**	Homecare needs	Help with activities of daily living (ADLs), including bathing, dressing, eating, toilet use, and meal preparation Help with additional in-home services such as occupational therapy and nursing care Homecare services to support independent living
	Practical needs	Transportation to medical appointments and errands Nutritious food and food security Stable housing Affordable housing Managing medication Feeling safe in home and neighborhood Help managing finances Financial assistance for healthcare and homecare costs Improving financial security/stability
**Information needs**	Health information needs	Improving knowledge about chronic health conditions Improving health literacy Improving medication knowledge
	Support service information needs	Where to find available social services Improving knowledge about social services
**Technology needs**	Technology use needs	Need for assistive technology (AT) Help with using technology Availability of health monitoring technologies
	Device development needs	Need for simple and reliable hardware Need for enjoyable and interactive elements in technology
**Healthcare system needs**	Healthcare access needs	Access to healthcare services, including specialists, dental care, and mental health professionals Ability to refill prescriptions and health supplies
	Healthcare provider training needs	Culturally competent care for ethnic and sexual minorities Specializations in treating mental health in older adults Physicians with geriatric medicine training Primary care physicians with knowledge about cognitive health

*Note*. The unmet needs identified in the literature search are reported by thematic domain and subtheme.

### Health management needs

Physical, cognitive, and mental needs were reported in 23 articles: 10 original needs assessments^6–15^ and 13 prior research.^16–28^ The literature identified three main themes. First, *physical health needs* addressed the needs related to physical aspects such as physical assistive devices and aids, which can improve well-being. Second, *cognitive and mental health needs* encompassed management of depression, sleep, memory problems, and cognitive health. Finally, *chronic condition management needs* involved managing symptoms of chronic illness and pain.

#### Physical health needs

Older adults reported physical health and well-being as unmet needs.^7^^,^^9^^,^^14^^,^^15^^,^^17^^,^^19^^,^^20^^,^^24^^,^^25^ Assistance with mobility and physical activity stimulation was reported as a need.^7^ Older adults stated that physical frailty increases the risk of needing long-term care.^17^ In the literature, older adults expressed concerns about oral pain and tooth loss.^14^^,^^20^^,^^24^ They also mentioned needing support for mobility, such as assistive walking devices, and accommodations for eyesight and hearing.^7^^,^^9^^,^^19^^,^^27^^,^^28^ Additionally, older adults indicated that support for physical activity is important for maintaining their health.^15^^,^^19^^,^^25^ Having adequate sleep and rest was an unmet need.^11^ The original needs assessment literature identified unmet needs in mobility support, eyesight and hearing, oral health, and sleep.^7^^,^^9^^,^^11^^,^^14^^,^^15^

#### Cognitive and mental health needs

Older adults reported issues with memory problems and the need for mental health services and informal support.^6^^,^^25^ They also mentioned needing psychological counseling to support mental health.^10^^,^^26^ Maintaining good mental health and self-esteem was highlighted as crucial for their overall well-being.^11^ Original needs assessments identified memory and cognitive support as unmet needs.^6^^,^^7^

#### Chronic condition management needs

Older adults identified managing chronic illnesses as an unmet need.^8^^,^^13^^,^^16^^,^^19^^,^^23^ Older adults reported challenges in obtaining adequate care for chronic conditions, which in turn limited their quality of life.^8^^,^^19^ The burden of symptoms associated with chronic illnesses, encompassing physical, psychological, and social frailty, was highlighted as a major concern.^8^^,^^13^ Health issues, including pain management, breathlessness, visual and hearing impairments, urinary problems, and fatigue, were also unmet needs.^16^^,^^23^ Effective pain control and strategies for falls prevention, fatigue relief, and depression relief, especially when co-occurring with pain, were essential for improving the well-being of older adults.^23^ Original needs assessment articles found unmet needs related to managing chronic conditions, including symptom burden, chronic pain, managing frailty, and chronic condition medication management.^8^^,^^10^^,^^12^^,^^13^

### Social needs

Unmet social needs in older adults were identified in 21 articles: 15 original needs assessments^6–9^^,^^11^^,^^29–38^ and six prior research.^16^^,^^18^^,^^39–42^ Two subthemes were identified among the unmet social needs: social connections and emotional support needs. *Social connection needs* identified the needs related to the size and satisfaction of older adult social networks and the opportunities available for socialization. *Emotional support needs* were related to the opportunities to speak about feelings with others, improving self-esteem, and coping with feelings of dependency and hopelessness.

#### Social connection needs

A few articles identified feeling socially isolated and lonely as a major unmet need in older adults.^16^^,^^18^^,^^39^ Lack of social contacts and dissatisfaction with the quality of social contacts contributed to reported feelings of loneliness and social isolation.^16^^,^^39^ Some older adults reported not having friends or family to turn to for help.^31^^,^^36^^,^^39^ Chronic illness was found to limit older adults’ ability to be socially active.^35^ Positive social interactions with family and friends led older adults to report less feeling of loneliness, while negative social interactions greatly contributed to loneliness.^7^ Providing support for others with reciprocity in social relationships and feeling connected to their community improved social satisfaction among older adults.^39^ A feeling of belonging was identified as a need.^11^ Lack of opportunities for socialization was also reported.^9^^,^^18^^,^^32^^,^^34^^,^^38^^,^^39^^,^^41^ Poor public infrastructure and a lack of locations for socialization were also identified as limitations to socialization.^35^ Older adults also endorsed needing social contacts to help gain health information and navigate complex care systems.^42^ In the original needs assessment literature, older adults reported a need for more socialization, more family support, and more social integration in the community.^6^^,^^9^^,^^31^^,^^32^^,^^34–38^

#### Emotional support needs

Emotional support needs related to older adults aging in place, not having opportunities to discuss their emotions or receive care from their social networks, for emotional distress. Feelings of worry, hopelessness, and despair were reported among older adults, but few reported having social connections with whom they could discuss these feelings.^29^^,^^33^ Older adults reported not having social contacts from whom they could receive emotional support.^40^ Older adults endorsed wanting social contacts to discuss their feelings.^30^ Lack of coping skills for emotional needs was also identified.^6^ Older adults endorsed wanting interventions that provide emotional support.^7^ Emotional support for managing chronic illness was also identified as an unmet need.^8^ Additionally, the need for positive self-esteem was identified.^11^ Older adults reported needing the community to cope with feelings of increased dependency on others.^37^ Increasing self-esteem and having someone to speak to about their emotions and to provide emotional support were reported in the original needs assessments.^7^^,^^8^^,^^11^^,^^29^^,^^30^^,^^33^

### Homecare and practical needs

The literature search identified homecare and practical needs in 25 articles: 13 original needs assessments^7^^,^^10^^,^^29^^,^^30^^,^^32^^,^^33^^,^^36^^,^^43–48^ and 12 prior research^19^^,^^21^^,^^22^^,^^26–28^^,^^49–54^. Two subthemes were identified: *homecare needs* related to supporting independent living and help with activities of daily living (ADLs), and *practical needs* related to logistical and pragmatic needs such as transportation, food availability, and managing finances.

#### Homecare needs

Services to provide help around the home were identified as an unmet need for older adults. Activities of daily living (ADL) limitations and the need for help with bathing, dressing, toileting, mobility, and eating were prevalent among older adults with and without disabilities.^7^^,^^22^^,^^26–28^^,^^43^^,^^47^^,^^49^^,^^52^^,^^54^ Unmet needs with instrumental activities of daily living (IADLs), such as using the telephone, shopping for groceries, preparing meals, taking medication, and handling money, were found.^7^^,^^22^^,^^26–28^^,^^45^^,^^48^^,^^53^ Having help in the home for ADLs and IADLs, including support for independent living, was reported in original needs assessments.^7^^,^^33^^,^^43^^,^^45^^,^^47^^,^^48^

#### Practical needs

Unmet practical needs for older adults were reported in the literature. The need for general transportation services or public transportation access was identified.^27^^,^^44^^,^^53^ For example, the ability to travel to healthcare appointments through available rides or public transit was reported as a specific need.^29^ Additionally, access to nutritious foods and a healthy diet was an unmet need.^10^^,^^21^^,^^32^^,^^33^^,^^44^^,^^47^^,^^51^^,^^53^ Food security was an unmet need, particularly for older adults with mental illness and during the COVID-19 pandemic.^19^^,^^46^ A need for assistance and support with end-of-life planning was reported.^30^ Older adults faced challenges in accessing affordable and accessible housing.^44^ The need for feeling safe in one’s home neighborhood was also identified as an unmet need.^53^ Feelings of safety also highlighted the need for age-friendly environments, such as those that prevent falls.^36^ Older adults reported the need for nutrition and meal services, transportation, and stable and affordable housing in the original needs assessments.^10^^,^^29^^,^^32^^,^^36^^,^^44^^,^^46^^,^^47^

Older adults reported finances as an area of unmet need. They described feeling insecure about having enough retirement funds and their ability to manage finances as well as uncertainty about their financial future.^27^^,^^36^^,^^50^ The ability to pay for healthcare costs was reported as an issue.^29^ Older adults struggled with eligibility for financial assistance for homecare services and the ability to pay for homecare services.^21^ The ability to find employment to supplement funds was a difficulty for older adults.^36^ Financial security and financial support for medical expenses were reported in the original needs assessments.^29^^,^^36^

### Information needs

Information needs were identified in seven articles: six original needs assessments^30^^,^^31^^,^^33^^,^^34^^,^^44^^,^^45^ and one prior research.^42^ The information needs were subcategorized as *health information needs* for self-management of chronic conditions and *support service information needs* to identify available services in the community.

#### Health information needs

Information needs regarding chronic condition management and health information were unmet for older adults. Information on safe medication use to improve self-management of chronic conditions in older adults was also an unmet need.^33^ Information on memory care services and mental health was also unmet.^30^^,^^34^^,^^45^ These needs were also highlighted in the original assessment literature.^30^^,^^33^^,^^45^

#### Support service information needs

Where to find support services was an unmet need for older adults. Older adults reported having insufficient knowledge about available social services, such as meal services, mental health services, and transportation services, as well as being unable to find information about such services.^31^^,^^42^^,^^44^ Older adults wanted to know how to learn about social services ­available within the community. All identified support service information needs were represented in the original needs ­assessments. ^31^^,^^34^^,^^44^^,^^45^

### Technology needs

Technology needs were identified in seven articles, all of which were original needs assessments.^55–61^ Needs regarding help with technology use and technology literacy were classified as *technology use needs*. Technology design requirements for an older adult audience were categorized as *device development needs*.

#### Technology use needs

Older adults reported needing help using technology.^55–60^ Articles suggest that older adults are open to using technology but face barriers such as limited technology skills, unfamiliarity with technology, and high costs. ^55–60^ There is a need for assistive technology (AT) to compensate for decreased strength and energy.^58^ Older adults reported the desire for assistive technologies to assist with ADLs and IADLs and improve safe living in the home, such as jar openers, wheeled laundry carts, emergency alert systems, and electronic agendas.^58^^,^^59^ AT devices enhance the quality of life, yet social stigma and self-esteem issues hinder their use.^58^ Addressing these barriers is essential for improving the adoption and effectiveness of AT. Older adults also reported wanting digital interventions developed for improving physical activity, memory, psychological well-being, and nutrition.^61^

#### Device development needs

A few articles also identified needs related to developing devices for older adults. One article suggested that older adults prioritize practicality and ease of use over aesthetic features in technology.^55^ Their main concerns include reliable hardware, simple operation processes, and readability.^55^ Devices created with older adult end-users in mind were an unmet need.^56^ Devices often do not meet the usability needs of older adults.^57^ When platforms are designed for older adults with chronic health conditions, the incorporation of enjoyable and interactive elements is essential for increasing user engagement.^57^ Social interaction within these platforms can significantly improve the overall user experience, as reported.^57^

### Healthcare system needs

Unmet needs related to accessing healthcare systems were identified in 17 articles: 12 original needs assessments^8^^,^^10^^,^^36^^,^^45^^,^^46^^,^^62–68^ and five prior research.^19^^,^^21^^,^^24^^,^^42^^,^^69^ Unmet needs were related to *healthcare access needs* such as availability of healthcare services such as health clinics and appointments available in the community as well as *healthcare service quality needs*, outlining limitations in the care that is provided.

#### Healthcare access needs

Older adults reported needing improved access to healthcare services. Improved availability of dental, hearing, and vision services, such as increased appointment availability and an increased number of services available in the community, was requested.^19^^,^^24^^,^^65^^,^^66^ The availability of pain management and palliative care services was an unmet need.^8^ Gaps were reported in access to homecare services to help with nutritional needs and mental health needs.^21^ Older adults reported having unmet needs accessing medical and mental health services.^10^^,^^36^^,^^69^ The availability of medical supplies and medication refills during the COVID-19 pandemic was a large unmet need for older adults.^46^ Older adults reported needing help navigating the complex healthcare system.^42^ Original needs assessments reported a lack of healthcare access in rural areas, availability of services for managing chronic conditions, including oral and mental health, and access to medical supplies as unmet needs.^8^^,^^10^^,^^36^^,^^46^^,^^65^^,^^67^

#### Healthcare service quality needs

Gaps were identified in the healthcare quality provided to older adults by healthcare professionals. Older adults felt as if they did not spend enough time with their primary care providers (PCPs) and felt they could not discuss memory issues and cognitive health with their PCPs.^45^^,^^64^ Healthcare providers also need training to better identify mental health issues in older adults, as depression in older adults is often overlooked.^63^ Finally, culturally informed care is needed for understanding the unique health needs of ethnic and sexual minorities.^62^^,^^67^^,^^68^ LGBT older adults reported not feeling comfortable discussing their healthcare needs with their PCPs.^62^^,^^68^ Language barriers among ethnic minorities also limit access to healthcare.^67^ Original needs assessments identified needs for culturally informed care, providing better mental health screening, and the ability of PCPs to manage geriatric health conditions.^45^^,^^62–64^^,^^67^^,^^68^

## Discussion

This systematic review sought to identify unmet needs of older adults aging in place. The unmet needs we identified spread across six theme domains: health management needs; social needs; homecare and practical needs; information needs; technology needs; and healthcare system needs. Homecare and practical needs and health management needs were the most reported unmet needs in the reviewed literature (25 articles and 23 articles, respectively). An unmet need was often defined by the inability to independently perform ADLs or IADLs and endorsed through surveys or questionnaires. Defining an unmet need by the ability to perform ADLs may have influenced the number of articles reporting unmet homecare needs. When focusing only on the original needs assessments, social needs were reported in the most articles (15 articles). When examining the literature on unmet needs, it is important to consider how unmet needs are defined and who is identifying the unmet need: self-reported by older adults or aggregated by researchers.

The literature identified a wide variety of unmet needs domains, highlighting the multifaceted nature of older adult unmet needs. Although this systematic review did not focus specifically on subpopulations, we discovered that individuals with intersectional identities, such as ethnic minority or sexual minority older adults with chronic health conditions, experienced compounded unmet needs.^10^^,^^31^^,^^43^^,^^48^^,^^62^^,^^67^ This surfaces a need to better understand the context of the individual when addressing unmet needs. Personalized solutions are needed to address complex, overlapping unmet needs as compared to one-size-fits-all solutions.

The systematic review identified a broad range of unmet needs affecting older adults. These needs highlight key areas where innovative approaches are needed to improve the quality of life and promote healthy aging. Next, we discuss how novel technology can be leveraged to address these diverse challenges and support aging populations more effectively. Although traditional interventions have often addressed these needs in isolation, advances in artificial intelligence (AI), such as large language models (LLMs) and natural language processing (NLP), can enable integrated, novel, person-centered technological solutions.^70^^,^^71^

### Health management needs and technology

AI-enabled tools can support older adults in managing their health through real-time monitoring, preventive interventions, and personalized guidance in the home. Wearables and smart home sensors integrated through the Internet of Things (IoT) allow for non-invasive tracking of vital signs, activity levels, and sleep patterns.^72^ AI systems analyze this data to detect anomalies, predict health risks, and provide tailored feedback to both older adults and caregivers. For example, AI-enabled fall detection systems and gait analysis tools can identify risk factors before a fall occurs, enhancing prevention efforts and promoting confidence in older adult mobility.^73^ Conversational agents can address chronic disease management by offering personalized reminders that also increase uptake and use of the technology.^74^

### Social needs and technology

AI-powered conversational agents can offer simulated companionship through responsive, natural dialogue.^75^ These tools can maintain continuity across sessions, personalize conversations, and provide a supportive and emotionally responsive environment.^76^ In the home setting, conversational agents have been shown to alleviate feelings of loneliness by providing consistent, low-effort interaction opportunities, especially for individuals with limited mobility or decreased social networks.^76^ These interactions can encourage older adults to engage more socially with others by providing reminders to contact loved ones.^77^

### Homecare and practical needs and technology

Smart home devices can control lighting, temperature, and appliances to create safer living environments, especially for individuals with mobility impairments or cognitive limitations.^77^ AI-driven technology can guide older adults through ADLs.^77^ Voice assistants like Amazon Alexa can help older adults complete IADLs by managing to-do lists, sending messages, booking transportation, and ordering groceries and other household essentials.^78^

### Information needs and technology

Many older adults experience difficulty in accessing health information related to chronic condition management. Conversational agents powered by LLMs can help bridge this gap by offering simple, easy to understand explanations of medical conditions and care instructions.^79^ AI chatbots can also direct users to appropriate community resources, such as mental health services, by interpreting natural language questions and providing localized recommendations.^80^ These features are particularly helpful for older adults with cognitive impairments or limited digital literacy.

### Technology usability needs

Older adults often cite technology usability issues and limited digital skills as major barriers to adoption.^77^ Many consumer devices are not designed with older adult usability needs in mind, which reduces their accessibility. LLM-powered conversational agents significantly reduce the need for manual navigation and allow users to interact through natural conversation, thus lowering the technological burden.^81^ Furthermore, stigma around visible assistive devices may be reduced by integrating these functions into common technologies like tablets and smart speakers to increase device uptake.^82^

### Healthcare system needs and technology

LLMs-powered interfaces (eg, chatbots, voice assistants, avatars) can be programmed to comprehend and respond to health-related inquiries and can offer immediate, conversational access to medical information, including medication reminders and initial diagnostic guidance, increasing healthcare access.^83^ For those with language barriers, multilingual NLP systems integrated into telemedicine can translate interactions.^84^ The use of conversational agents may improve healthcare access for diverse populations.

Overall, this literature review identified unmet needs of older adults. It was strengthened by using a variety of sources, including both original needs assessments and secondary analyses, across three databases. The focus on older adults in the home adds to the literature of unmet needs in older adults. This systematic review has some limitations. Literature published prior to the year 2013 was not included in the literature. This limitation is mitigated by including systematic reviews scoped with research articles from earlier years. Literature included in this review was also limited to the three databases included. Articles not included in the databases were not available for review and therefore were unable to be included in this systematic review.

Future work in unmet needs for older adults can focus on creating novel AI and technology solutions to address current societal needs beyond those identified in the systematic review. For example, LLMs could be created to address digital literacy and assist older adults with digital tasks such as sending emails, online banking, and e-shopping in a secure manner. Additionally, online security could be enhanced for older adults targeted by fraudulent schemes. New directions for LLMs could help in fraud detection to protect older adults in the digital community. These future directions highlight the transformative potential of AI and LLMs in not only addressing existing unmet needs but also proactively safeguarding and empowering older adults in an increasingly digital world.

## Conclusion

We conducted a systematic review of the literature to identify unmet needs for older adults aging in place. We contributed a categorization of the unmet needs into six domains: health management needs; social needs; homecare and practical needs; information needs; technology needs; and healthcare system needs. We found that the unmet needs of older adults are wide-ranging and provide novel targets for new technologies. Finally, we discussed how the recent advances in AI technologies have the potential to address many of the unmet needs. Overall, we see many promising venues for technology to better support aging-in-place.

## Supplementary Material

igaf106_Supplementary_Data

## Data Availability

The data underlying this article are available in and can be accessed as the database of the systematic review literature and extracted needs available publicly as supplementary material.
