# Supplementary material for: A systematic review of unmet needs of older adults in home settings and their implications for novel technological solutions
Source: Innov Aging. 2025 Oct 10;9(Suppl 1):S14–23. doi: 10.1093/geroni/igaf106 (PMC12742851; doi:10.1093/geroni/igaf106)
Supplement: igaf106_Supplementary_Data [file igaf106_supplementary_data.docx]

***Innovation in Aging* Supplementary Material: Dolman et al. A Systematic Review of Unmet Needs of Older Adults in Home Settings and Their Implications for Novel Technological Solutions.**

**Database Queries:**

*PubMed:*

("older adult*"[tiab] OR elder*[tiab] OR Aging[tiab]) AND ("Evidence Gaps"[Mesh] OR "Needs Assessment"[Mesh] OR "needs assessment*"[tiab] OR "unmet need*"[tiab]) NOT ("Residential Facilities"[Mesh] OR facilit* OR "nursing home" OR "assisted living" OR Hospitals[Mesh])

*CINAHL:*

((MM "Aged") OR (MM "Aged, 80 and Over") OR (MM "Frail Elderly") OR (MM "Adult Care (Saba CCC)") OR AB "older adult*") AND ((MM "Needs Assessment") OR(MM "Self Assessment") OR (MM "Human Needs (Psychology)") OR (MM "Human Needs (Physiology)") OR (MM "Information Needs") OR (MM "Evidence Gaps")) NOT ((MH "Residential Facilities+") OR (MH "Nursing Homes+") OR (MH "Assisted Living"))

*ProQuest:*

(MAINSUBJECT.EXACT("Centenarians") OR MAINSUBJECT.EXACT("Oldest old people") OR MAINSUBJECT.EXACT("Elder care") OR MAINSUBJECT.EXACT("Aging") OR MAINSUBJECT.EXACT("Older people") OR MAINSUBJECT.EXACT("Aging in place") OR MAINSUBJECT.EXACT("Frailty")) AND ((MAINSUBJECT.EXACT("Needs analysis") OR MAINSUBJECT.EXACT("Needs")) OR summary(“need* assessment*”) OR summary(“unmet need*”)) NOT ((MAINSUBJECT.EXACT("Assisted living facilities") OR MAINSUBJECT.EXACT("Nursing homes") ) OR summary("Residential Facilit*") )
